# Fluorescence-Guided Surgery to Detect Microscopic Disease in Ovarian Cancer: A Systematic Review with Meta-Analysis

**DOI:** 10.3390/cancers17030410

**Published:** 2025-01-26

**Authors:** Evrim Erdemoglu, Carrie L. Langstraat, Amanika Kumar, Stuart A. Ostby, Marlene E. Girardo, Andrea Giannini, Kristina A. Butler

**Affiliations:** 1 Department of Medical and Surgical Gynecology, Mayo Clinic, Phoenix, AZ 85054, USA; erdemoglu.evrim@mayo.edu; 2 Department of Gynecologic Oncology, Suleyman Demirel University, Isparta 32260, Turkey; 3 Department of Obstetrics and Gynecology, Division of Gynecologic Surgery, Mayo Clinic, Rochester, MN 55905, USA; 4 Department of Biostatistics, Mayo Clinic, Phoenix, AZ 85054, USA; 5 Division of Obstetrics and Gynecology, Department of Clinical and Experimental Medicine, University of Pisa, 56126 Pisa, Italy; andrea.giannini@unipi.it

**Keywords:** fluorescence-guided surgery, ovarian cancer, OTL38, 5-ALA, peritonectomy, EC17, ovarian neoplasm

## Abstract

**Background**: The objective in epithelial ovarian cancer is to reach maximal cytoreduction with no visible residual tumor. Tumor detection during cytoreductive surgery depends on visual inspection, palpation, or blind biopsy, methods that lack reliability for identifying microscopic disease. Although the importance of microscopic disease in epithelial ovarian cancer is controversial, it may harbor chemoresistant cells and explain the high recurrence rates. Fluorescence-guided surgery (FGS) is an emerging approach. However, the potential in ovarian cancer remains underexplored; the majority of the existing evidence pertains to gastrointestinal tumors and a limited group of ovarian cancer patients. Their comparative effectiveness is still uncertain. **Objective:** To systematically review and evaluate the role of fluorescence-guided surgical techniques in detecting microscopic disease in ovarian cancer and compare their efficacy to total peritonectomy. **Data Sources:** A systematic search was made in three databases (PubMed, Web of Science, and Embase). The search was conducted from 1975 to 2024, including randomized controlled trials, observational studies, and conference abstracts in the last 25 years. **Study Selection:** Clinical studies published in English involving ovarian cancer patients undergoing FGS or total peritonectomy were included. Case reports, reviews, animal studies, and studies involving mixed cancer populations without ovarian cancer-specific data were excluded. Two independent reviewers screened 631 studies, yielding 12 eligible studies for final analysis. **Data Extraction and Synthesis:** Data were extracted and synthesized in accordance with PRISMA and MOOSE guidelines, using random-effects models for independent analysis. Sensitivity, specificity, positive predictive value (PPV), and odds ratios (ORs) were grouped, accompanied by subgroup analyses based on the fluorescence agent employed. For quality assessment, we utilized the NIH quality tool. **Main Outcome(s) and Measure(s):** The primary outcome was the rate of change in surgical management due to fluorescence guidance or total peritonectomy. Secondary outcomes comprised lesion-level sensitivity, specificity, and PPV. Safety outcomes included adverse events associated with fluorescence agents. **Results:** There were 12 studies involving 429 ovarian cancer patients. FGS improved the detection of microscopic disease compared to standard visualization methods, with a pooled sensitivity of 0.77. Folate receptor-targeted agents had high sensitivity (84%) but low specificity (26%). Aminolevulinic acid (5-ALA) showed superior diagnostic accuracy with a sensitivity of 84% and a specificity of 96%. Total peritonectomy showed no significant advantage over FGS for detecting microscopic disease. The adverse events were mild, with no serious events reported. We observed a high heterogeneity across studies and methodologies. **Conclusions and Relevance:** Fluorescence-guided surgery utilizing fluorescence tracers demonstrates potential in improving the detection of microscopic disease and may change surgical management in epithelial ovarian cancer, particularly with 5-ALA. Variability in performance and limited data on survival outcomes necessitates additional research. Total peritonectomy does not offer further advantage in the detection of microscopic disease. Future trials should focus on standardizing methodology and evaluating the effects of microscopic disease removal on survival outcomes. **Registration:** The study was registered to PROSPERO as CRD42024578274.

## Simple Summary

The identification of tumors during cytoreductive surgery relies on visual inspection, palpation, or blind biopsy methods that exhibit limited reliability in detecting microscopic disease. Fluorescence-guided surgery (FGS) utilizes various tracers for the identification of microscopic disease. The objective of this study was to evaluate the efficacy of FGS in the detection of microscopic disease through meta-analyses, while discussing the existing evidence and potential implications for ovarian cancer. The pooled odds ratio of a change in surgical plan/microscopic disease for FGS, standard techniques, and peritonectomy was 1.29 and 1.14, respectively. Among the FGS tracers, folate receptors exhibited high sensitivity; however, their low specificity resulted in false positives. 5-Aminolevulinic acid exhibited high sensitivity (84%) and specificity (96%) in detecting microscopic disease. Standardized protocols and phase III trials are essential to determine the role of FGS in ovarian cancer. We discuss the significant variability in existing studies, the limitations of FGS, and the potential implications for future research.

## 1. Introduction

The performance of maximal cytoreductive surgery (CRS) is the standard-of-care surgical approach in ovarian cancer whether performed primarily or after neoadjuvant chemotherapy [1]. Historically, maximal CRS depends upon the naked eye to determine macroscopic tumor volume with changing goals for cytoreduction over time [2,3,4,5,6]. At present, the goal is complete gross resection to achieve no residual disease, but despite this, 70–80% of ovarian cancer patients ultimately experience recurrence and most frequently of the peritoneum [7,8,9,10]. With the advent of poly-ADP ribose polymerase (PARP) enzyme inhibitors such as olaparib, niraparib, and rucaparib, and the use of bevacizumab, survival was improved particularly in patients with BRCA mutation or homolog repair deficiency [11,12]. Recurrence is reduced to <70% with PARPi use in patients with mutation [10,13,14,15,16]. Therefore, some hypothesize that there is merit in moving beyond assessment with the naked eye alone for disease resection and suggest either total peritonectomy or use of fluorescent tracers to guide selective peritonectomy [17].

Currently, neither approach for the treatment of microscopic disease has been evaluated against standard treatment. When performed, total peritonectomy is defined as a resection parietal peritoneum from nine compartments: the right and left subphrenic regions, Morrison’s pouch, the right and left paracolonic regions, the right and left pelvic walls, overlying the bladder, and the pouch of Douglas. Ovarian cancer may also metastasize to the porta hepatis, and it can be incorporated into total peritonectomy [18,19]. Nonetheless, following total peritonectomy, peritoneal recurrences still occur. Fluorescence-guided surgery (FGS) is currently being explored to tailor surgical therapy and assist in meaningful performance of partial peritonectomy by aiding the detection and removal of microscopic disease. Additionally, FGS may assist in early-stage ovarian cancer by improving upon random peritoneal biopsies to identify microscopic disease associated with worse prognosis and resulting in accurate staging [20]. Finally, at the time of interval CRS, chemotherapy can obscure even macroscopic disease and make detection less sensitive due to inflammation, scarring, or treatment effects. Gadduci et al. assessed the failure and survival of ovarian cancer patients with microscopic residual disease after second-look surgery. They reported that 85% of patients developed recurrence and the most common sites of recurrence were the abdomen and pelvis. This study indicated a worse outcome in patients with microscopic residual disease after first-line chemotherapy [9].

Various tracers with different pathophysiological mechanisms have been proposed for real-time visualization of metastatic tumors are gaining popularity in neurosurgery, thoracic surgery, and surgical oncology. Indocyanine green (ICG), pafolacianine (OTL38, Cytalux^®^ On Target Laboratories West Lafayette, Indiana, USA), EC17, 5-aminolevulinic acid (5-ALA), and pegsitacianine have emerged as promising agents in fluorescence-guided surgery. ICG binds to plasma proteins and emits fluorescence when excited by light at 820–840 nm [21]. ICG is most commonly used to identify the sentinel node in uterine, cervical, and vulvar cancer [22]. OTL38 is a fluorescent tracer designed by On Target Laboratories that binds to folate receptor-alpha (FRα), which is overexpressed in ovarian cancer. It contains a cyanine dye and emits at wavelengths of 774–794 nm [23]. EC17 targets FRα like OTL38 but contains fluorescein which is in the visible wavelength: 490–530 nm [23,24]. Aminolevulinic acid (5-ALA) is a new agent that has been widely adopted in glioblastoma neurosurgery to enhance tumor resection with reduced false positives [25]. It is metabolized to protoporphyrin IX which is a photosensitizing agent. 5-ALA fluoresces when exposed to blue light. Under blue light, the final product protoporphyrin IX emits violet fluorescence at 620–710 nm [26]. Pegsitacianine is a pH-sensitive agent conjugated to the indocyanine green [27]. The selective binding of these agents to tumoral tissue and emitting light at near infrared wavelengths provides a good tumor-to-background differentiation and is the basis of identification of lesions not visible to the naked eye [28]. OTL38 was recently approved by the Food and Drug Administration (FDA) for use as an intraoperative imaging agent for ovarian and lung cancer. 5-aminolevulinic acid (5-ALA; Gleolan^®^ Photonamic GmbH and Co. KG, Pinneberg, Germany) is approved by the FDA for intraoperative imaging in high-grade glioma surgery [29].

Although the concept of cytoreduction has evolved from 2 cm to no residual disease [30], the value of additional surgical effort to remove microscopic disease is controversial. Disagreement over the best management of microscopic disease to both prevent and eradicate chemoresistant cells remains ongoing. The role of additional surgical effort to remove microscopic disease is controversial. Current systematic review data for FGS in ovarian cancer combines findings for gastrointestinal cancers and animal studies which may misrepresent the performance of FGS for detection of microscopic disease in ovarian cancer. In the present systematic review, we compare total peritonectomy to FGS for selective peritonectomy during CRS for ovarian cancer. Furthermore, we aim to analyze sensitivity, specificity, and positive predictive value (PPV) of FGS in ovarian CRS.

## 2. Materials and Methods

Study reporting is in accordance with the Preferred Reporting Items for Systematic Reviews and Meta-Analyses (PRISMA 2020) reporting guidance [31] and systematic reviews of observational studies (MOOSE) checklists [32]. Study registration is through PROSPERO as CRD42024578274.

### 2.1. Eligibility Criteria

Clinical trials with a randomized controlled, quasi-experimental design or non-randomized single-arm studies published in English were eligible. We also evaluated published conference abstracts. Case reports, reviews, commentaries, letters, opinions, and studies with a mixed population of various cancers and that did not respond were excluded. Ex vivo and animal studies were excluded.

### 2.2. Search Strategy

We searched PubMed, Web of Science, and Embase systematically between 31 July and 7 August 2024. The references of appropriate articles and any relevant systematic reviews identified were used for backwards and forward citation searching on PubMed.

The screening process included articles between 1975 and 2024 and finally processing articles in the last 25 years. The MeSH keywords used for the fluorescence imaging agents search were “pafolacianine”, “indocyanine green”, “aminolevulinic acid”, and “spectroscopy, near-infrared”, or the non-MeSH keywords “ONM100”, “EC-17”, “EC17”, and “pegsitacianine”. Each of these MeSH/free text keywords were combined using the Boolean ”AND” operator with MeSH keywords including “ovarian neoplasms” or “carcinoma, ovarian epithelial”, “cytoreduction surgical procedures”, and the free text term “ovarian tumor”. There was no MeSH identified for total peritonectomy; the free text terms (peritonectomy) OR (total peritonectomy), (residual disease) OR (microscopic ovarian cancer)), OR (microscopic residual disease) were combined using the Boolean AND with the MeSH term “ovarian neoplasms” or “carcinoma, ovarian epithelial”, or “cytoreduction surgical procedures”.

### 2.3. Selection Process

Two independent investigators screened all studies identified in our search by titles and abstracts for eligibility. Disagreements were resolved in a panel meeting including three authors. The results of the search were managed by Covidence [Covidence Systematic review software, Veritas health Innovation, Melbourne, Australia]. The Covidence version was accessed on 30 July 2024 after transferring as an XML file by Endnote (The EndNote Team. Version EndNote X9 or EndNote 20. Philadelphia, PA, USA: Clarivate, 2013). Duplicate records were removed.

### 2.4. Data Extraction

Full-text analyses of the articles were performed by E.E. and A.G. to retrieve articles that fulfilled the eligibility criteria under the PICOS framework. (P) Population: women with primary or recurrent ovarian cancer undergoing surgery (including primary, interval or secondary debulking, and second-look procedures); [I] Intervention: intra-operative use of any of the fluorescence-emitting agents OTL38 (pafalocianine), EC17, OTM100 (pegsitacianine), 5-ALA, or ICG; [C] Comparison: naked eye visualization under white light or palpation which is the standard of care; [O] Outcome: Main critical outcome 1: additional cancer on tissue not planned for resection and not detected by white light/palpation that changed surgical management, or the removal of microscopic disease. Main outcome 2; sensitivity, specificity, false-positive and false-negative rate, and PPV calculated at the lesion level. Secondary outcome; treatment-emergent adverse events and serious adverse events; [s] Studies: clinical trials (randomized controlled trials or observational studies). Studies that do not fulfill the PICOS criteria, case reports, reviews, commentaries, letters, opinions, and studies with a mixed population of various cancers and that did not respond were excluded.

### 2.5. Outcome Measures and Critical Outcomes; Were Categorized into Three Sets

(A) Patient-level analysis: proportion of ovarian cancer patients that had a change in surgical plan after fluorescent tracer application; primary critical outcome. A change in surgical plan was defined as any additional procedure added to the originally planned surgery based on the detection of microscopic disease, not identified by visual inspection or palpation.

(B) Lesion-level analysis; sensitivity, specificity, PPV, false-positive rate, and false-negative rate were secondary outcomes.

(C) Treatment-emergent adverse events and serious adverse events/mortality.

### 2.6. Measures of Treatment Effect

The actual involvement of peritoneum and other sites in terms of microscopic disease is unknown because the standard-of-care assessment is made by naked eye visual inspection and palpation. In the absence of a clear effect size, we reviewed the literature to determine the lower and upper limits for the effect size of the critical main outcome. There are few studies reporting the pathologic evidence of microscopic disease in macroscopically normal-appearing peritoneum [33]. In contrast, a recent study in which multiple peritoneal biopsies per patient were taken from macroscopically normal peritoneum identified microscopic disease in 26.9% of patients [34]. Based on this, we selected a threshold of 30% for a meaningful threshold with the assumption that the prevalence of microscopic disease is probably underestimated owing to the large discrepancy between studies and the fact that the entire peritoneal specimen may only undergo limited processing during pathologic assessment. The prevalence of microscopic disease is probably underestimated because the surface area of the peritoneum is comparable to the body surface area and the entire surface of the peritoneum cannot be evaluated. Moreover, for a threshold of 20–30%, we considered the main outcomes as having undetermined clinical significance and <20% as insignificant [34,35,36]. For this study, we accepted a sensitivity of 64% for visual examination as similarly reported in the VIPER study for threshold analysis [37].

### 2.7. Unit of Analysis Issues

Studies included in this meta-analysis are single-arm studies with observations on participants. The unit of analysis was individual patients and lesions resected. To ensure consistency, patient-level data and lesion-level data from studies were extracted to 2 × 2 tables and the available outcomes were recorded.

### 2.8. Risk of Bias and Quality Assessment

The studies were assessed using the National Institute of Health (NIH) quality assessment tool for pre-post, non-randomized, single-arm interventional trials. We assessed the risk of reporting bias using funnel plots and Egger’s test to detect publication bias. Additionally, we used the Outcome Reporting Bias in Trials (ORBIT) tool to evaluate selective outcome reporting.

### 2.9. Statistical Analysis

We performed a meta-analysis of the included studies using a random-effects model. Studies were excluded if they did not include an outcome in each intervention group or did not have enough information required for proportional data comparison. We pooled the point estimates of OR from each study using the generic inverse-variance method of Der Simonian and Laird. The heterogeneity of effect size estimates across these studies was quantified using the I^2^ statistic. The I^2^ statistic ranges in value from 0 to 100% (I^2^ < 25%, low heterogeneity; I^2^ = 25–50%, moderate heterogeneity; and I^2^ > 50%, substantial heterogeneity). Subgroup analyses were performed if the heterogeneity is moderate or substantial to explore the source of heterogeneity. A *p*-value of <0.05 was considered significant. All data analyses were performed using the R version 4.2.2 using meta and metafor package 3.

## 3. Results

**Figure 1:**
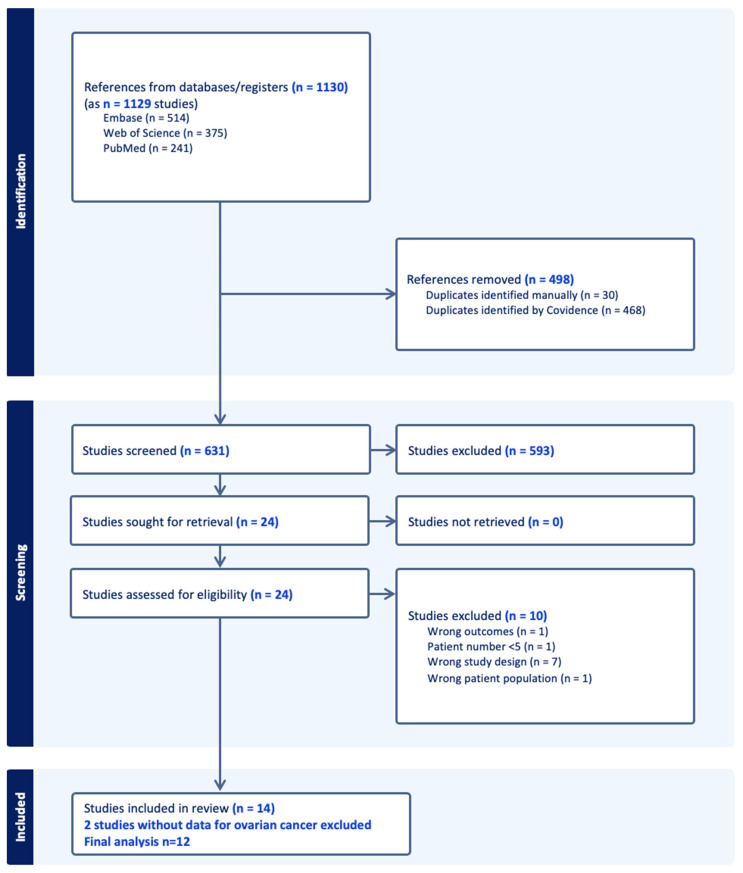
PRISMA Flow Diagram of Study Search and Selection. After removal of duplicates, 631 studies were screened out of 1130 studies. Of the eligible studies 14 studies, 2 did not provide data regarding ovarian cancer, and finally 12 studies were included in this meta-analysis.

A total of 1130 results were identified, and 631 records were uploaded to Covidence after removing duplications. Of the 25 records assessed for eligibility, 14 articles were included in our final review. Figure 1 shows the PRISMA flow of the articles. Finally, two papers were removed [38,39] because their results were mainly comprised of colorectal cancer and did not provide data for a separate ovarian cancer analysis. Analysis was completed in 12 publications (Figure 1) [24,40,41,42,43,44,45,46,47,48,49] including 1 poster presentation [50]. The study characteristics and types of interventions are shown in Supplementary Table S1.

### 3.1. Primary Critical Outcome

**Figure 2:**
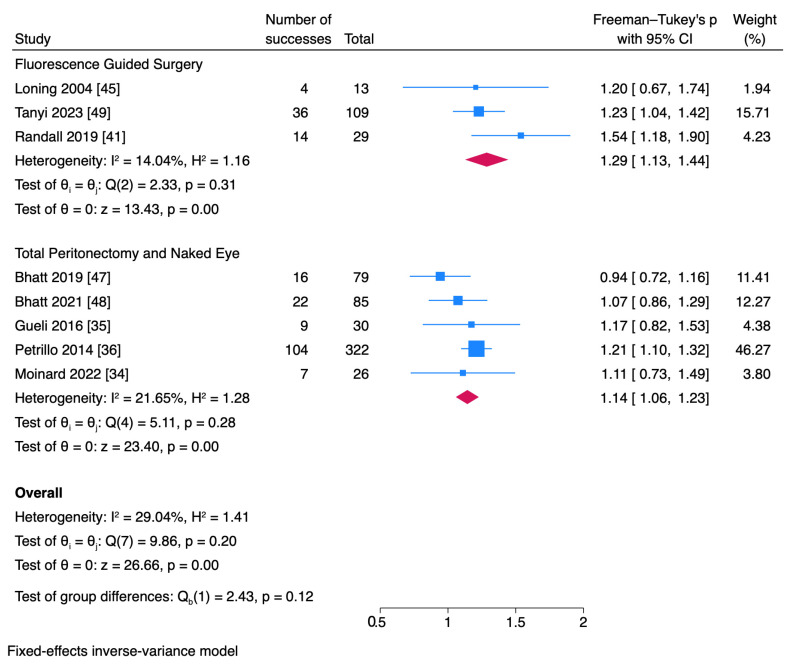
Fluorescence-guided surgery vs. the naked eye. Microscopic disease that caused a change in surgical plan or microscopic disease after peritonectomy and standard of care. Forest plot for odds ratio for changes in surgical plan based on microscopic disease detection is given. The pooled odds ratio for FGS, standard techniques, and peritonectomy was 1.29 and 1.14, respectively. The heterogeneity was low [34,35,36,41,45,47,48,49].

**Figure 3:**
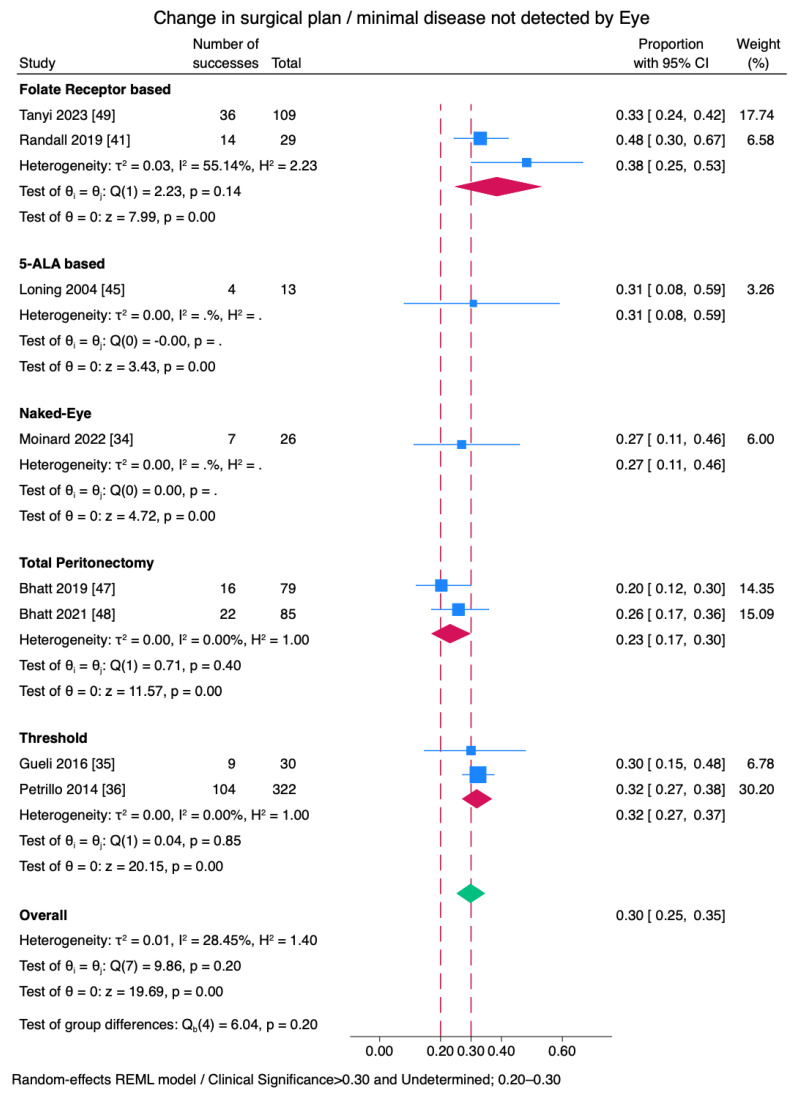
Subgroup divisions for each methodology. Microscopic disease that caused a change in surgical plan or microscopic disease after peritonectomy and standard of care. Performance, odds ratio and 95% confidence intervals, and heterogeneity are displayed [34,35,36,45,47,48].

Only 3 studies reported a change in surgical management. The number of articles reporting microscopic disease in healthy-appearing peritoneum after total peritonectomy or naked eye assessment were 2 and 1, respectively. The results are provided in a forest plot for a visual summary; we approached the pooled effect and intergroup comparison with caution due to the limited number of studies. The odds ratio of a change in surgical plan based on microscopic disease was 1.29 for fluorescence-guided surgery. The heterogeneity across the group was I^2^: 14%. The pooled odds ratio for a change in surgical plan for folate receptor studies was 1.29 (95% CI 1.13–1.44). The odds ratio for total peritonectomy was 0.94–1.07 (*p* > 0.05). Three studies [6,7,8] were used as benchmarks for determining the threshold. The odds ratio of these thresholds was 1.11–1.21 (Figure 2 and Figure 3).

### 3.2. Secondary Outcomes

**Figure 4:**
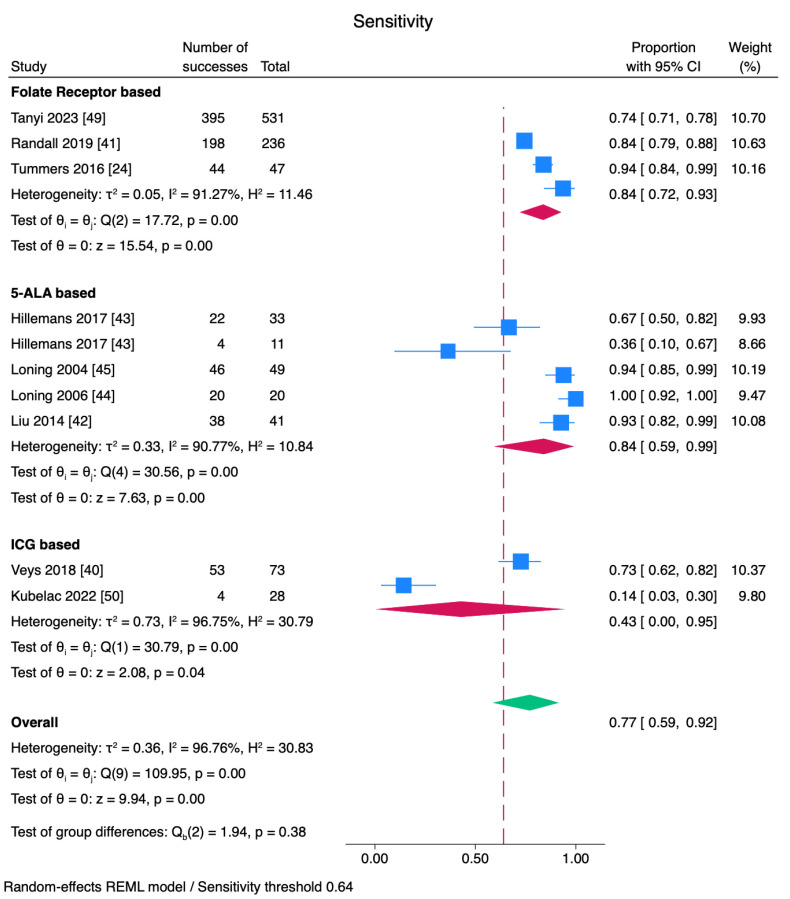
Sensitivity analyses for FGS. Sensitivity estimates for each tracer are displayed. The overall sensitivity of FGS was 77%. Folate receptor agents and 5-ALA showed higher sensitivity rates with substantial heterogeneity [24,40,41,42,43,44,45,49,50].

**Figure 5:**
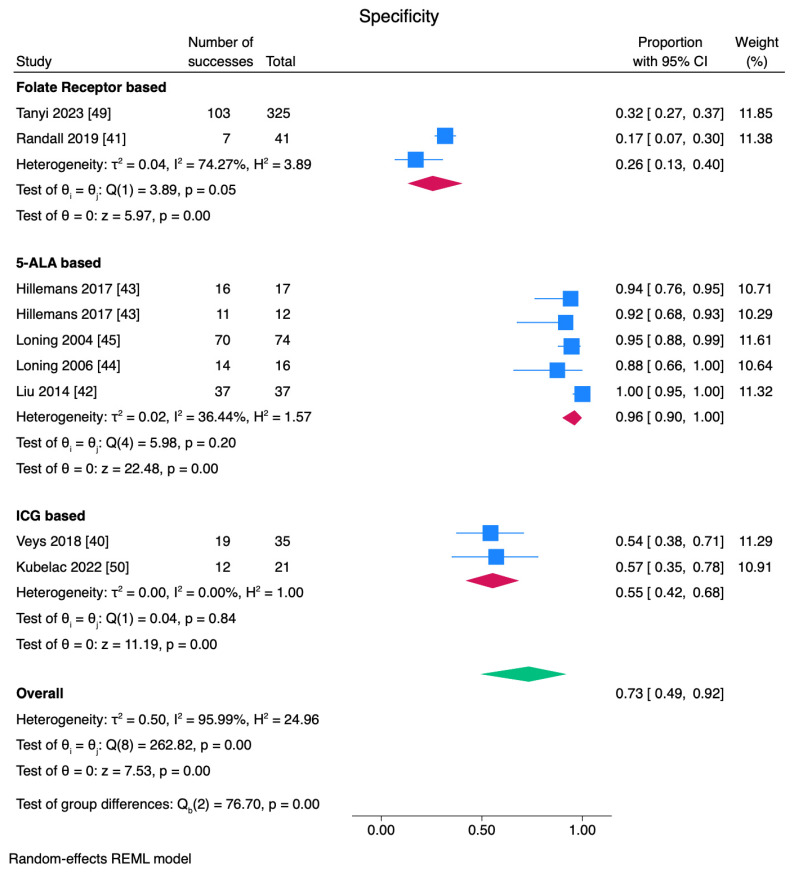
Specificity analyses for FGS. 5-ALA reached the highest specificity to detect true negatives, with low heterogeneity. Folic acid receptor agents had lower specificity, and studies displayed a higher variability for folic acid receptor agents [40,41,42,43,44,45,49,50].

**Figure 6:**
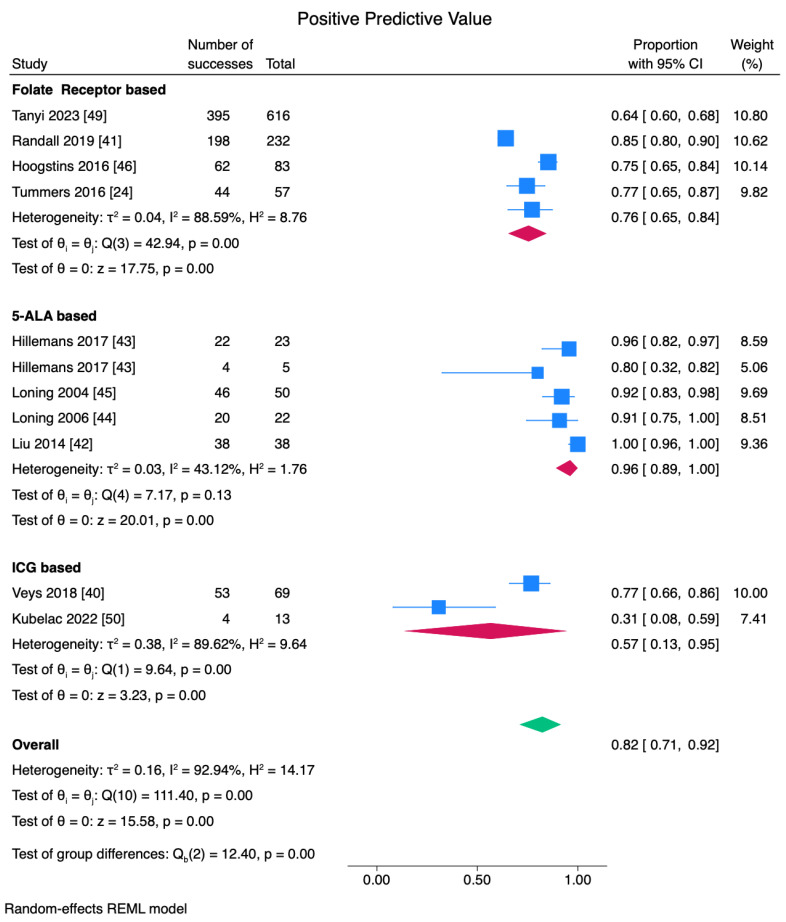
PPV analyses for FGS. 5-ALA showed higher reliability in detecting true positives with 96% PPV. Whereas folic acid receptor tracers exhibited moderate PPV (76%) [24,40,41,42,43,44,45,46,49,50].

Sensitivity, specificity, and PPV are shown in Figure 4, Figure 5 and Figure 6. While sensitivity was similar across all agents (*p* = 0.38), specificity and positive predictive value were significantly different among subgroups (*p* < 0.01). A combined sensitivity of 0.77 was found for fluorescence surgery. In subgroup analysis by tracer type, folate receptor-based agents were found to have a pooled sensitivity of 84% but varied across studies (I^2^: 91.2%). 5-ALA-based agents had a sensitivity of 84% with variability across studies (I^2^: 90.77%) (Figure 4).

The pooled specificity of 0.26 for folate receptor agents with a high variability indicates these agents have a low ability to detect true negatives and the false-positive rate is high. Variability across the studies indicates more studies are needed. ICG had a moderate pooled specificity of 0.55 with minimal heterogeneity across studies. In contrast to OTL38 and ICG, 5-ALA had a pooled estimate of 0.96 for specificity. We find the result to be reliable as the heterogeneity across studies was low (Figure 5).

False-positive and false-negative rates are shown in Supplementary Table S1. The pooled PPV values for folate receptor agents, 5-ALA, and ICG are 0.76, 0.96, and 0.57. The PPV results for OTL38 and ICG were highly variable across studies (Figure 6).

5-ALA seems to be the best agent in terms of sensitivity, specificity, and positive predictive value with lower heterogeneity across studies; however, the difference across study methodologies and lacking information on changes in surgical plan opens room for further investigation.

### 3.3. Meta-Regression

In meta-regression analyses, we investigated if increasing the number of biopsies changed the diagnostic values. The number of biopsies per patient was not found to be an important factor in determining sensitivity, specificity, false-positive and false-negative values, or microscopic disease detected by fluorescence.

### 3.4. Safety Assessment

Studies show that fluorescence tracers using the folate receptor have an acceptable safety profile with the most common side effects including nausea, vomiting, and abdominal discomfort. The events are usually mild to moderate. No drug-related serious adverse events are reported, as shown in Table S1. There have been no adverse effects reported for 5-ALA or ICG; however, we suspect reporting bias and limited data.

### 3.5. Quality of Data and Publication Bias

The quality of data is shown in Supplementary Table S2. The quality of data in 8 studies was fair and 1 study was poor. The remaining 3 studies were good. The Egger test indicated publication bias for false positives, specificity, PPV, and microscopic disease. However, we detected no bias for sensitivity analysis.

## 4. Discussion

### 4.1. Microscopic Disease Detection and the Role of Fluorescence-Guided Surgery in Ovarian Cancer

The use of FGS improved the detection of microscopic disease by 31–48% which did meet our critical threshold set at 30% for clinical significance. In select reference studies, the incidence of microscopic residual disease after primary or interval cytoreduction was approximately 30%. Microscopic disease was identified more often using FGS than total peritonectomy. While FGS, particularly with folate receptor tracers and 5-ALA, has provided more clinical benefits compared to others based on our defined reference clinical values, there is variability between studies, and not all fluorescence agents perform effectively. To reduce the variability in FGS, the trace dosage and administration needs to be standardized and the performance studied.

### 4.2. Sensitivity, Specificity, Positive Predictive Value, and False-Positive Rate

While fluorescent tracers showed high sensitivity and a positive predictive value, the specificity and false-positive rates varied. All methods except ICG had a sensitivity higher than the threshold value of 64%. ICG seems less reliable compared to other agents. The high variability for each tracer may be attributed to differential dosing and routes of administration (intraperitoneal, intravenous, or oral). The high heterogeneity in diagnostic values indicates the effectiveness may vary and more studies are needed.

Notably, the body of research on fluorescence tracers in ovarian cancer remains limited, with most data derived from gastrointestinal and mixed cancer populations. Study heterogeneity across study methodologies applies to our findings in meta-regression: the number of biopsies per patient did not affect sensitivity, specificity, or the detection of microscopic disease. More standardized methodologies should be used in FGS research.

Our findings indicate a high false-positive rate for folate receptor tracers compared to other agents. It is essential for surgeons to be aware of the pitfalls and the existing gaps in specificity. Although FRα is overexpressed in up to 90% of ovarian cancers, including high-grade serous and endometrioid subtypes, it is less prevalent in low-grade and clear cell carcinomas [51,52]. Despite its design to target FRα, OTL38 cross-reacts with folate receptor beta (FRβ), which is predominantly expressed in immune cells, such as tumor-associated macrophages, rather than in the cancerous epithelium [53]. This cross-reactivity leads to false-positive fluorescence in benign lymph nodes, as observed in both ovarian and lung cancers [53,54,55]. The higher rates of false positives and lower specificity may result in irrelevant resections, consequently anticipated to augment morbidity. We were unable to find any information in the existing literature concerning the increased morbidity and extended duration of surgical procedures attributable to false positive results. The advancement of novel tracers and the enhancement of existing ones is feasible in future research, which would focus on the implications and causes of false positives.

Intra-op detection of occult ovarian carcinoma using an FRα-specific fluorescent ligand EC17 study (NCT01511055) was terminated following technical problems with the investigational camera system. OTL38 (NCT04941378) aimed to compare the performance of different camera imaging systems in assessing the positive predictive value and sensitivity to detect folate-positive ovarian cancer using the gold standard of pathologic review. This study was withdrawn due to a sponsor request in 2022. A pilot and feasibility study of EC17 in the intraoperative detection of occult ovarian carcinoma (NCT02000778) is completed but the results are not published. The surgical use of folate receptor agents has not been superior, and ongoing trials have been terminated. Despite this, there have been many registered studies investigating the role of targeted systemic treatment utilizing folate receptors in ovarian cancer.

ICG has demonstrated low sensitivity and a high false-positive rate. A current trial (NCT05633836) is enrolling patients to detect microscopic disease using ICG during diagnostic laparoscopy for ovarian cancer prior to CRS. This study aims to evaluate the effect of ICG on assessing resectability. Another objective is to evaluate the presence of microscopic disease through FGS after cytoreduction.

5-ALA, commonly used in brain tumors, is emerging as a promising tracer for ovarian cancer with a high sensitivity and high specificity. OVA-302, a phase III study (NCT05804370), is actively recruiting patients to investigate the real-time detection of epithelial ovarian cancer during cytoreductive surgery. Upon administration, 5-ALA accumulates in malignant cells and is metabolized to fluorescent protoporphyrin IX (PpIX), though it may also be present in non-tumor tissues. The required metabolism eliminates fluorescence in necrotic tissue. Compared to OTL38, 5-ALA demonstrates a lower false-positive rate, although it too may highlight inflammatory or reactive tissues.

### 4.3. Peritonectomy

The probability of microscopic residual disease following primary or interval CRS is significant [56]. The study undertaken by Majd et al. [57] assessed radical upper abdominal surgery, with a specific focus on diaphragm stripping and resection, with the objective of attaining complete cytoreduction. The surgical intervention was carefully planned based on preoperative imaging results and a comprehensive macroscopic examination of the peritoneal surface conducted during laparoscopy, which was later confirmed at the laparotomy phase. In the final evaluation of specimens, 4% of patients demonstrated no disease evidence. Furthermore, 4% of individuals in the neoadjuvant cohort exhibited solely microscopic disease. It is significant that a restricted cohort of patients demonstrating pleural involvement in histological examination exhibited macroscopic disease during the surgical procedure; therefore, even thoracoscopy might be insufficient to diagnose pleural disease. The documented rate of microscopic disease-free margins ranged from 86% to 92%. This and other studies indicate that even the aggressive surgical approach might be futile in attempts to achieve complete cytoreduction. The overall survival rates were similar between diaphragmatic peritonectomy and diaphragmatic full-thickness resection with pleurectomy (51% versus 48% at 35 months of follow-up). Visual inspection alone is insufficient for detecting microscopic disease, and the evaluation of the neoadjuvant chemotherapy response remains suboptimal. New methods such as FGS may help to close this gap and may help to stratify patients better compared to the naked eye or laparoscopy.

Macroscopic tumor assessment is employed to stratify patients during primary cytoreduction and interval debulking surgery. However, the reliability of such results was questionable; although a higher laparoscopic index was proposed as a predictive factor for incomplete cytoreduction, the risk of unnecessary laparotomy remained at 33% [8,18]. GOG182 [56] developed a model involving disease score, stage, CA-125, ascites, and stage–age interaction for the purpose of selecting patients for cytoreductive surgery. The disease score (DS) reflected the anatomical extensivity of disease: (1) DS-low, characterized by pelvic and retroperitoneal dissemination; (2) DS-moderate (DS-mod), indicating further spread to the abdomen while sparing the upper abdomen; and (3) DS-high, denoting upper abdominal involvement impacting the diaphragm, spleen, liver, or pancreas. However, changes in the surgical plan and the extensivity of microscopic disease by utilizing FGS warrants further investigation regarding their impact on these scoring systems.

Recent studies highlight the critical need for the prospective validation of surgical interventions in ovarian cancer. A previous study [58] showed that the assessment of macroscopic tumor spread, prior to and following neoadjuvant chemotherapy, quantified by the peritoneal cancer index (PCI) and its change (Δ-PCI), demonstrates a notable correlation with the histopathological response and the attainment of complete cytoreduction. Observations of macroscopic changes in tumor burden indicate that a Δ-PCI cut-off value of ≥15 was reported to be a reliable predictor of complete cytoreduction (R0), a chemotherapy response score (CRS) of 3, and improved disease-free survival. While disease-free survival was similar in patients that required visceral mesentery resection and the control, the benefits of extensive surgery in the retrospective design cannot be argued due to study limitations. The importance of moving forward from minimal residual disease to complete resection with no detectable disease in patients with ovarian cancer has been clearly established. The median overall survival range extended from 106 months for individuals with a complete resection to 34 months for those with residual disease exceeding 2 cm. The investigation carried out by Tozzi et al. involved the execution of mesenteric debulking with the objective of attaining complete cytoreduction [17]. The occurrence of widespread, disseminated disease affecting the mesenteric root required a standardized systematic approach, resulting in segmental intestinal ischemia in a group of patients.

Microscopic foci, particularly chemoresistant cell populations, may contribute to disease relapse [6,20]. Although the removal of macroscopic disease is known to improve survival [59], there are no studies assessing the impact of removing microscopic disease on long-term outcomes. FGS offers a potential advantage over total peritonectomy with lower morbidity, less time, and more individualized surgical care. Prospectively designed studies are needed to evaluate its effects on disease-free survival and overall survival.

The detection of microscopic disease by total peritonectomy was not superior in our analyses. The detection rate of microscopic disease in a total peritonectomy specimen does not necessarily increase solely because of its comprehensive nature. Detection is influenced by the sampled sites and the methods employed for pathological analysis. This aligns with the sentinel lymph node biopsy concept, as complete lymphadenectomy does not increase metastasis detection rates compared to targeted sentinel lymph node evaluation. This underscores the significance of strategic sampling and thorough pathological examination in improving diagnostic accuracy. In addition, future studies should focus on reducing heterogeneity and investigating the long-term impact of removing microscopic disease on survival outcomes.

A phase I and II study investigating the efficacy and safety of peritonectomy and visceral–peritoneal resection to achieve complete cytoreduction yielded that resection of mesentery was required in 20% of stage IIIC–IV ovarian cancer patients. The study concluded that the addition of mesentery resection provided complete cytoreduction in 100% of patients [17]. Although this study highlights the surgical techniques used to complete cytoreduction, it underscores the microscopic disease. The use of FGS in such a study might lead to the detection of extensive disease in more than 20% of patients. The utilization of FGS in such a study would help to reduce morbidity by providing more precise surgical results. It might also challenge the current concept of residual disease leading us to redefine the boundaries of complete cytoreduction.

### 4.4. Safety

Although the sensitivity and positive predictive value of tracers are emphasized, less attention is given to their limitations and potential side effects, particularly for tracers like 5-ALA in ovarian cancer, which require further investigation in prospective trials. 5-ALA has been safely administered to thousands of brain cancer patients. The OTL38 safety profile is better established, yet data on other tracers remain scarce and possibly biased due to underreporting. Within the included studies, adverse effects were consistent across the tracers, with no serious adverse event reported. Safety data are limited, low quality, and based on small sample sizes. Long-term follow-up is also missing. Larger trials with extended monitoring periods are required to validate the safety and efficacy.

## 5. Conclusions

In summary, while FGS, particularly folate receptor-targeted and 5-ALA tracers, may improve the detection of microscopic disease and contribute to CRS, the variability of tracer performance must be considered. FGS may help to further study the relation between microscopic disease removal and survival. Without histological proof of microscopic disease, it was not possible to identify the importance of removal of microscopic disease, particularly when patients receive adjuvant chemotherapy. Currently, there is no high-quality data to support the benefits of fluorescence agents and total peritonectomy in ovarian cancer. Hence, more data on disease-free survival and overall survival are needed to support the benefit of this technology or total peritonectomy in ovarian cancer. Revisiting the findings of study of Bristow et al. cytoreduction with no gross macroscopic residual disease increases the overall survival [59], further studies evaluating the role of resecting microscopic disease is required. Although it is proposed that peritonectomy with hyperthermic intraperitoneal chemotherapy, or use of FGS in various tumors could increase oncological efficacy [60,61,62,63,64,65], its applicability to ovarian cancer remains uncertain. A recent published randomized controlled trial proposed that progression-free survival might be better in patients where more microscopic disease is removed, the follow-up is short, and results are preliminary (ChiCTR1800018771) [66].

There is also a paucity of information on early-stage ovarian cancer and fluorescent tracers. Prior studies do not include early-stage ovarian cancer. However, the most beneficial effect of a tracer with high sensitivity and low false positivity might be helpful to detect microscopic disease in apparent stage I ovarian cancer. Rather than perform random biopsies of the peritoneum, FGS may assist in microscopic disease detection and in some cases replace lymphadenectomy and attendant morbidity. Detecting microscopic disease in early-stage ovarian cancer requires a great surgical effort by comprehensive staging involving pelvic and paraaortic lymphadenectomy [67]. The comprehensive lymphadenectomy procedure is associated with morbidity, along with an increased duration of the operation and length of hospital stay [68]. The high false-positive rate of FGS in lymph nodes is significant not only in the early stages but also in advanced stages. In the LION trial [69], a total of 647 women with normal-appearing lymph nodes were randomly allocated to receive complete cytoreductive surgery, either with or without pelvic and para-aortic lymph node dissection. The final pathology assessment revealed positive lymph nodes in 55.7% of patients who underwent lymph node dissection; however, no survival advantage and increased morbidity was seen. False positivity in lymph nodes may be due to drainage of the antibody and might be associated with the tracer type and dose [70]. The false-positive rate of FGS, particularly OTL38, was highest in lymph nodes when compared to other anatomical sites [39,41,46]. This is one of the issues to be addressed and examined in both translational research and clinical trials. Henning et al. reported that distant metastatic microscopic lesions can be overlooked in early-stage ovarian cancer. They questioned cardiophrenic lymph node metastasis as one of the first presentations in high-grade serous carcinomas that will later evolve into transperitoneal disease [71]. The implementation of FGS in the early stage of ovarian cancer could improve our understanding of disease dissemination and assist in identifying atypical metastasis that may not be observable to the naked eye. Tognon et al. reported high grade, stage IB, IC, and IIA, positive cytology, age greater than 54 years, dense adhesions, and specific histology were poor prognostic factors [67]. They argued that dense adhesions and positive cytology are associated with specific molecular characteristics. These adhesions harboring microscopic tumors with particular molecular alterations might be easily missed during standard surgical care. FGS may close this gap, accurately stage the patient, and identify tumor cells with specific molecular alterations. FGS has the potential to identify contralateral involvement in early-stage ovarian cancer and may aid in tailoring wedge resection/cystectomy for fertility preserving. Another potential of FGS could be in restaging of patients who have had an incomplete staging surgery for early ovarian cancer.

The benefits of FGS in various histologic types have not been studied, and the detection of microscopic disease might be redundant for some histological types. The standard care of surgery and peritonectomy might be justified for low-grade serous ovarian cancer; the extent of cytoreduction is not correlated well with overall survival compared to high-grade serous ovarian cancer [72]. The threshold of cytoreduction is macroscopic: 2.5 mm for low-grade serous [73].

While FGS was shown to be promising in detecting the extensivity and volume of disease, the molecular signatures and tumor biology seem to be unknown. Recent studies have indicated that epithelial-to-mesenchymal transition in ovarian cancer is related to the peritoneal spread and platin resistance of tumors [74,75,76]. Hypoxia-related miRNAs, e-cadherin, and other epithelial-to-mesenchymal transition markers are seen as targets for therapy [30,76,77]. These molecular signatures might be the cause of failure despite complete cytoreduction. Future incorporation of these molecular signatures to FGS may help to identify resistant tumor cells, paving the way to a more targeted surgical approach.

More robust phase III trials are needed to assess the safety and diagnostic efficacy of tracers, like 5–ALA. We also propose that increased surgical complexity and operative time should be considered. This will help to analyze whether the promising diagnostic benefits are offset by the unreported or rare adverse effects. Some tracers are more promising compared to others for further investigation for ovarian cancer. The addition of fluorescent tracers to the current standard of care may be considered to optimize outcomes. However, both methods are currently experimental and based on limited quality data. Theoretical advantages of adding fluorescent tracers to the standard of care over total peritonectomy could be decreased surgical complexity, a reduced learning curve, and decreased morbidity.

## Supplementary Materials

The following supporting information can be downloaded at https://www.mdpi.com/article/10.3390/cancers17030410/s1, Table S1: Diagnostic features, administration details of FGS agents and adverse events; Table S2: Quality Assessment of Included Studies Using the NIH Tool.
