# Supplementary material for: Fluorescence-Guided Surgery to Detect Microscopic Disease in Ovarian Cancer: A Systematic Review with Meta-Analysis
Source: Cancers (Basel). 2025 Jan 26;17(3):410. doi: 10.3390/cancers17030410 (PMC11815761; doi:10.3390/cancers17030410)
Supplement: Supplementary file 1 [file cancers-17-00410-s001.zip › cancers-3392754-supplementary.pdf]

Supplementary Materials: Fluorescence-Guided Surgery to Detect Microscopic Disease in Ovarian Cancer: A Systematic Review with Meta-Analysis

Evrim Erdemoglu, Carrie L. Langstraat, Amanika Kumar, Stuart A. Ostby, Marlene E. Girardo, Andrea Giannini and Kristina A. Butler

Table S1. Diagnostic features, administration details of FGS agent, adverse events. Study characteristics including route and dose of FGS agent are variable across studies. Patient number (n) is given for primary analysis. Some studies have more patients in the safety evaluation. TEAE: treatment emergent adverse events, SAE: severe adverse events, AESI: adverse events of special interest.

		n	*Dose*	*Timing before surgery*	*Route*	*Total Number of biopsies*	*Biopsy per patient*	*False Positive*	*False Negative*	*TEAE*	*SAE*	*Tracer related*
Folate Receptor	Tanyi 2023 [1]	109	0.025mg/kg OTL38	<4-9 h	IV	855	7.84	68% (63-73%	26% (0.22-0.29)	Not given	Event rate given/ patient number not given.	30% (45/150)
	Randall 2019 [2]	29	0.025mg/kg OTL 38	<4-9 h	IV	277	9.5	83% (70-93%)	16% (12-21%)	100% (44/44	15.9% (7/44)	18.2% (8/44)
	Hoogstins 2016 [3]	12	0.0125-0.05mg/kg OTL 38	<4-9 h	IV					82.7% (24/29)	0% (0/29)	96.5% (28/29)
	Tummers 2016 [4]	12	0.1 mg/kg EC17	ICG based	IV					80% (12/15)	0%	46.7% (7/15)
5-ALA	Hillemans 2017 [5]	15	10mg/kg	<4-9 h	PO	50	3.3	6% (5-24%)	33% (18-50%)	None	None	
	Hillemans 2017 [5]	5	10mg/kg	>9h	PO	23	4.6	8% (7-32%)	64% (33-90%)	None	None	
	Loning 2004 [6]	12	30mg/kg	<4-9 h	IP	123	10.25	5% (1-12%)	6% (1-15%)	None	None	
	Loning 2006 [7]	17	36mg/kg	<4-9 h	IP	36	2.11	12% (0-34%)	0% (0-8%)	Not given	Not given	
	Liu 2014 [8]	19	20mg/kg	<4-9 h	PO	78	4	0 (0-5%)	7% (7-18%)	None	None	
ICG	Veys 2018 [9]	20	0.25mg/kg	<4-9 h	IV	108	5.4	46% (0.29-0.62%)	27% (18-38%)	None	None	
	Kubelac 2022 [10]	15	Not provided	0		49	3.26	43% (0.22-0.65%)	86% (70-97%)	Not given	Not given	
Total Peritonectomy	Bhatt 2019 [11]	79	-	-	-	Total peritonectomy				-	-	
	Bhatt 2021 [12]	85	-	-	-	Total peritonectomy				-	-	

Table S2. Quality Assessment of Included Studies Using the NIH Tool. NIH Tool for quality assessment of manuscripts included in the study. The NIH assessment tool can be reached from Study Quality Assessment Tools | NHLBI, NIH

	Q1	*Q2*	*Q3*	*Q4*	*Q5*	*Q6*	*Q7*	*Q8*	*Q9*	*Q10*	*Q11*	*Q12*	*Q ASS*

Bhatt 2019 [11]	YES	YES	YES	YES	NOT	YES	NOT	NOT	YES	YES	NOT	YES	FAIR
Bhatt 2021 [12]	YES	YES	YES	YES	NOT	NOT	NOT	NOT	CD	NOT	NOT	YES	FAIR
Löning 2004 [6]	YES	YES	YES	YES	CD	YES	NOT	NOT	CD	YES	NOT	YES	FAIR
Hoogstins 2016[3]	YES	NOT	YES	NOT	NOT	YES	YES	YES	CD	NOT	NOT	YES	FAIR
Kubelac 2022 [10]	NOT	NOT	YES	YES	NOT	NOT	NOT	NOT	CD	NOT	NOT	YES	POOR
Löning 2006 [7]	YES	YES	YES	YES	NOT	YES	YES	NOT	YES	NOT	NOT	YES	FAIR
Hillemanns 2017[5]	YES	YES	YES	YES	NOT	YES	YES	NOT	CD	NOT	NOT	YES	FAIR
Liu 2014 [8]	YES	YES	YES	YES	NOT	YES	YES	NOT	CD	YES	YES	YES	GOOD
Randall 2019 [2]	YES	YES	YES	YES	YES	YES	YES	YES	NOT	NOT	NOT	YES	GOOD
Tanyi 2023 [1]	YES	YES	YES	YES	YES	YES	YES	YES	YES	NOT	YES	NOT	GOOD
Tummers 2016 [4]	YES	YES	YES	YES	NOT	YES	YES	NOT	CD	NOT	NOT	YES	FAIR
Veys 2018 [9]	YES	YES	YES	YES	NOT	YES	YES	NOT	CD	NOT	NOT	YES	FAIR

NHI Quality Assessment Tool.

1. Was the research question or objective in this paper clearly stated?
2. Was the study population clearly specified and defined?
3. Was the participation rate of eligible persons at least 50%?
4. Were all the subjects selected or recruited from the same or similar populations (including the same time period)? Were inclusion and exclusion criteria for being in the study prespecified and applied uniformly to all participants?
5. Was a sample size justification, power description, or variance and effect estimates provided?
6. For the analyses in this paper, were the exposure(s) of interest measured prior to the outcome(s) being measured?
7. Was the timeframe sufficient such that one could reasonably expect to see an association between exposure and outcome if it existed?
8. For exposures that can vary in amount or level, did the study examine different levels of the exposure as related to the outcome (e.g., categories of exposure, or exposure measured as a continuous variable)?
9. Were the exposure measures (independent variables) clearly defined, valid, reliable, and implemented consistently across all study participants?
10. Was the exposure(s) assessed more than once over time?
11. Were the outcome measures (dependent variables) clearly defined, valid, reliable, and implemented consistently across all study participants?
12. Were the outcome assessors blinded to the exposure status of participants?
13. Was the loss to follow-up after baseline 20% or less?
14. Were key potential confounding variables measured and adjusted statistically for their impact on the relationship between exposure(s) and outcome(s)?

Quality Rating (Good, Fair, or Poor).

Rater #1 initials:
Rater #2 initials:
Additional comments (If POOR, please state why):

References

1. Tanyi, J.L.; Randall, L.M.; Chambers, S.K.; Butler, K.A.; Winer, I.S.; Langstraat, C.L.; Han, E.S.; Vahrmeijer, A.L.; Chon, H.S.; Morgan, M.A.; et al. A Phase III Study of Pafolacianine Injection (OTL38) for Intraoperative Imaging of Folate Receptor-Positive Ovarian Cancer (Study 006). *J. Clin. Oncol.* **2023**, *41*, 276–284.
2. Randall, L.M.; Wenham, R.M.; Low, P.S.; Dowdy, S.C.; Tanyi, J.L. A phase II, multicenter, open-label trial of OTL38 injection for the intra-operative imaging of folate receptor-alpha positive ovarian cancer. *Gynecol. Oncol.* **2019**, *155*, 63–68.
3. Hoogstins, C.E.; Tummers, Q.R.; Gaarenstroom, K.N.; de Kroon, C.D.; Trimbos, J.B.; Bosse, T.; Smit, V.T.; Vuyk, J.; van de Velde, C.J.; Cohen, A.F.; et al. A Novel Tumor-Specific Agent for Intraoperative Near-Infrared Fluorescence Imaging: A Translational Study in Healthy Volunteers and Patients with Ovarian Cancer. *Clin. Cancer Res.* **2016**, *22*, 2929–2938.
4. Tummers, Q.R.; Hoogstins, C.E.; Gaarenstroom, K.N.; de Kroon, C.D.; van Poelgeest, M.I.; Vuyk, J.; Bosse, T.; Smit, V.T.; van de Velde, C.J.; Cohen, A.F.; et al. Intraoperative imaging of folate receptor alpha positive ovarian and breast cancer using the tumor specific agent EC17. *Oncotarget* **2016**, *7*, 32144–32155.
5. Hillemanns, P.; Wimberger, P.; Reif, J.; Stepp, H.; Klapdor, R. Photodynamic diagnosis with 5-aminolevulinic acid for intraoperative detection of peritoneal metastases of ovarian cancer: A feasibility and dose finding study. *Lasers Surg. Med.* **2017**, *49*, 169–176.

6. Löning, M.; Diddens, H.; Küpker, W.; Diedrich, K.; Hüttmann, G. Laparoscopic fluorescence detection of ovarian carcinoma metastases using 5-aminolevulinic acid-induced protoporphyrin IX. *Cancer* **2004**, *100*, 1650–1656.
7. Löning, M.C.; Diddens, H.C.; Holl-Ulrich, K.; Löning, U.; Küpker, W.; Diedrich, K.; Hüttmann, G. Fluorescence staining of human ovarian cancer tissue following application of 5-aminolevulinic acid: Fluorescence microscopy studies. *Lasers Surg. Med.* **2006**, *38*, 549–554.
8. Liu, Y.; Endo, Y.; Fujita, T.; Ishibashi, H.; Nishioka, T.; Canbay, E.; Li, Y.; Ogura, S.; Yonemura, Y. Cytoreductive surgery under aminolevulinic acid-mediated photodynamic diagnosis plus hyperthermic intraperitoneal chemotherapy in patients with peritoneal carcinomatosis from ovarian cancer and primary peritoneal carcinoma: Results of a phase I trial. *Ann. Surg. Oncol.* **2014**, *21*, 4256–4262.
9. Veys, I.; Pop, F.C.; Vankerckhove, S.; Barbieux, R.; Chintinne, M.; Moreau, M.; Nogaret, J.M.; Larsimont, D.; Donckier, V.; Bourgeois, P.; et al. ICG-fluorescence imaging for detection of peritoneal metastases and residual tumoral scars in locally advanced ovarian cancer: A pilot study. *J. Surg. Oncol.* **2018**, *117*, 228–235.
10. Kubelac, P.; Catalin, V.; Pasca, A.; Muntean, M.; Gata, V.; Morariu, D.; Oradan, A.; Soritau, O.; Fischer-Fodor, E.; Balacescu, O.; Pop, B.; Fetica, B.; Achimas-Cadariu, P. Indocyanine green in near-infrared light for intra-operative imaging of residual ovarian cancer after neoadjuvant chemotherapy: Initial experience. Int. J. Gynecol. Cancer **2022**, 32(Suppl 2).
11. Bhatt, A.; Sinukumar, S.; Mehta, S.; Damodaran, D.; Zaveri, S.; Kammar, P.; Mishra, S.; Parikh, L.; Ranade, R.; Penumadu, P.; et al. Patterns of pathological response to neoadjuvant chemotherapy and its clinical implications in patients undergoing interval cytoreductive surgery for advanced serous epithelial ovarian cancer- A study by the Indian Network for Development of Peritoneal Surface Oncology (INDEPSO). *Eur. J. Surg. Oncol.* **2019**, *45*, 666–671.
12. Bhatt, A.; Bakrin, N.; Kammar, P.; Mehta, S.; Sinukumar, S.; Parikh, L.; Shaikh, S.; Mishra, S.; Mallaya, M.; Kepenekian, V.; et al. Distribution of residual disease in the peritoneum following neoadjuvant chemotherapy in advanced epithelial ovarian cancer and its potential therapeutic implications. *Eur. J. Surg. Oncol.* **2021**, *47*, 181–187.
